# Small extracellular vesicles: from mediating cancer cell metastasis to therapeutic value in pancreatic cancer

**DOI:** 10.1186/s12964-021-00806-y

**Published:** 2022-01-03

**Authors:** Wenjie Zhang, Juan Xing, Tian Liu, Jie Zhang, Zhujiang Dai, Huan Zhang, Daorong Wang, Dong Tang

**Affiliations:** 1 Clinical Medical College, Yangzhou University, Yangzhou, Jiangsu Province China; 2 Department of General Surgery, Institute of General Surgery, Clinical Medical College, Northern Jiangsu Province Hospital, Yangzhou University, Yangzhou, 225001 China

**Keywords:** Pancreatic cancer, sEVs, Pre-metastatic niches, Tumor microenvironment, Treatment

## Abstract

Pancreatic cancer is a highly malignant tumor and, is extremely difficult to diagnose and treat. Metastasis is one of the critical steps in the development of cancer and uses cell to cell communication to mediate changes in the microenvironment. Small extracellular vesicles (sEVs)-carry proteins, nucleic acids and other bioactive substances, and are important medium for communication between cells. There are two primary steps in sVEs-mediated metastasis: communication between pancreatic cancer cells and their surrounding microenvironment; and the communication between primary tumor cells and distant organ cells in distant organs that promotes angiogenesis, reshaping extracellular matrix, forming immunosuppressive environment and other ways to form appropriate pre-metastasis niche. Here, we explore the mechanism of localization and metastasis of pancreatic cancer and use sEVs as early biomarkers for the detection and treatment of pancreatic cancer.

Video Abstract

**Supplementary Information:**

The online version contains supplementary material available at 10.1186/s12964-021-00806-y.

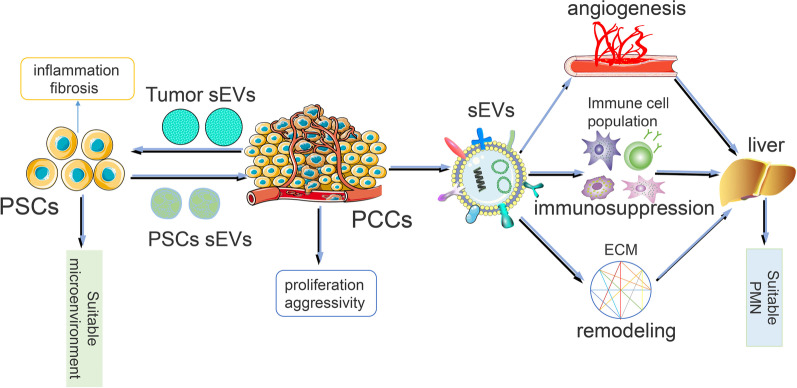

## Background

Pancreatic cancer is the seventh leading cause of cancer death in the world, with a rate of 2.5%, and a mortality rate of 4.5% in 2018.It usually has a poor prognosis, and the one-year and five year survival rate only 24% for a year and 9%,respectively [1, 2]. Despite recent advances in the treatment of pancreatic cancer and the discovery of new biomarkers in the early diagnosis of pancreatic cancer, no decline in the death rate of pancreatic cancer has been observed and it remains, one of the most deadly malignancies. Pancreatic cancer is difficult to treat, mostly because of the protective effect of the microenvironment outside the cancer cells, so the microenvironment of pancreatic cancer has been extensively researched. The microenvironment in pancreatic cancer is comprised of acellular stroma, cancer-associated fibroblasts (CAF, also known as pancreas stellate cells (PSC), immune cells, and soluble factors such as cytokines, chemokines, growth and pro-angiogenic factors [3].The microenvironment of pancreatic cancer plays an important role in tumor genesis, tumor development, metastasis, tumor immunosuppression, and chemotherapy resistance.

sEVs are nanosized vesicles that are actively secreted by almost all cells, including fibroblasts, endothelial cells, epithelial cells, neuronal cells, immune cells, and cancer cells [4]. They are enclosed by a lipid bilayer and carry various biomolecules, including proteins, glycans, lipids, metabolites, RNA, and DNA [5].The biogenesis pathway of sEVs depends on the endosomal sorting complex required for transport (ESCRT) for transportation, and its pathway is: early endosomes (EEs) are formed by the fusion of endocytic vesicles in the early stage, and the EEs are mostly transformed into LEs/ MVBs through the budding of membrane, and the required goods are packaged into ILVs. ILVs protein sorting can be ESCRT-dependent or independent. However, most of them are ESCET dependent and ubiquitinated substrates in the membrane part of the inner bud body. However, most of them are ESCET dependent and ubiquitinated substrates in the membrane part of the inner bud body. Ilv can be degraded in lysosomes or saved by DUB, while MVBs is guided by Rab27A and Rab27B to migrate to the periphery of the cell. Finally, the SNARE complex helps MVBs fuse with the plasma membrane, releasing ILVs into the extracellular lumens called exosomes [6, 7]. ESCRT-independent cargo loading into sEVs is mainly divided into lipid raft, ceramide and cargo sorting into sEVs. RNA also breaks into sEVs; RNA sorting into sEVs is unlikely to be random. Cellular abundance and miRNA, exo-motifs and miRNA sorting into sEVs [8]. sEVs play a key role in cell-to-cell communication. Pancreatic cancer cell-derived sEVs can be targeted to the distant organs through blood transportation, and can create an appropriate pre-metastatic niche for tumor metastasis by inducing angiogenesis, remolding extracellular matrix, and forming an immunosuppressive microenvironment in the distant sites [9].Tumor-sEV-mediated factors can also promote tumor initiation, metastasis, and therapy-resistance in cancer cells through cell–cell communication within the TME [10–12]. Other cell-derived sEVs associated with pancreatic tumor cells promote tumor proliferation, drug resistance, and metastasis. The pancreatic stellate cell-derived sEVs mediate information exchange between pancreatic cancer cells and pancreatic stellate cells, and can induce epithelial to mesenchymal transition (EMT) around pancreatic cancer cells and form a fibrotic microenvironment, which can prevent the entry of chemical drugs and promote the proliferation and metastasis of pancreatic cancer [13, 14]. sEVs secrete by tumor-associated macrophage (TAMs) has been shown to transfer miR-501-5p into PDAC cells, and down-regulate TGFbR3 by activating the TGF-β signaling pathway to promote the metastasis and invasion of PDAC cells [15].As a medium of communication between pancreatic cancer cells and other cells, sEVs play a crucial role in the development of pancreatic cancer regardless of cell derivatives.

This review aims to explore the biological significance of sEVs in the tumor microenvironment of pancreatic cancer. It focuses on the continuous change in sEVs in the tumor microenvironment and the distant microenvironment when pancreatic cancer cells migrate from the primary site to the distal organ. Our review also focuses on the application of sEVs in the treatment of pancreatic cancer.

## Two tunes of sEVs mediating pancreatic cancer metastasis

Metastasis is a key factor in tumor progression. It is a multi-step process and mainly consists of intercellular communication, including communication between tumor cells and the surrounding microenvironment and communication between tumor cells and cells in distant organs. sEVs are important mediums for cell- to-cell communication. Here, we explore the role of sEVs in pancreatic cancer metastasis, mainly the remodeling of the microenvironment around tumor cells and the formation of premetastatic niches, and highlight the mechanisms by which sEVs participate in these steps.

### Reshaping the microenvironment around cancer cells

**Fig. 1:**
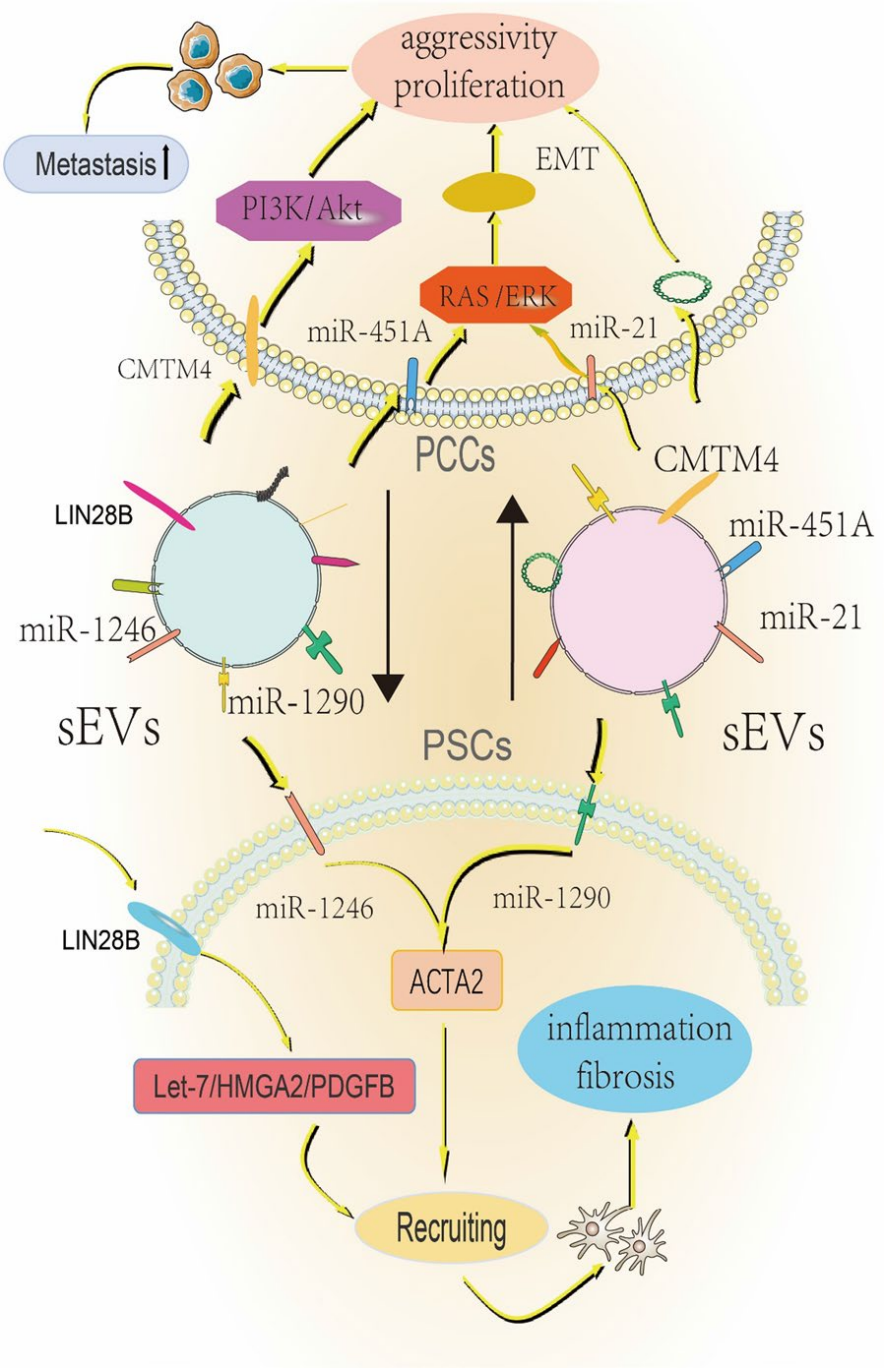
PCCS: Pancreatic cancer cells. PCCs and PSCs are based on the mechanism of interaction between sEVs. Pancreatic cancer-derived sEVs activate the LIN28B /let-7/HMGA2/PDGFB signaling pathway by expressing LIN28B, and recruit PSCs from afar to promote the formation of pre-metastatic niches. Secondly, the expression of miR-1246 and miR-1290 sEVs formed an inflammatory environment of niche by promoting the expression of PSCs fibrosis and other related genes. Conversely, miR-5703 expressed on PSCS-derived sEVs activated the PI3K/Akt pathway, miR-21 enhanced RAS /ERK signaling activity, and in anoxic environment: Both miR-4465 and miR-616-3p ultimately promoted the proliferation and metastasis of pancreatic cancer by inhibiting the PTEN/ Akt pathway

The microenvironment around pancreatic cancer tumor cells contains different cells and various other factors. Pancreatic stellate cells, which account for about 50% of the tumor stroma, play the most important role in remodeling the microenvironment around the tumor cells [16]. sEVs are involved in the communication between pancreatic stellate cells and pancreatic cancer tumor cells. Pancreatic stellate cells (PSCs) account for 4.7% of all pancreatic cells and have characteristics similar to those of stellate cells, including the accumulation of retinol esters in lipid vesicles and the ability to get activated [17, 18]. Pancreatic stellate cells and pancreatic cancer cells also interact with each other. Pancreatic stellate cells constitute the tumor matrix of pancreatic cancer and promote tumor growth and metastasis. Pancreatic cancer can also promote pancreatic stellate cell development and metastasis [19–21]. Activated pancreatic HSCs can be found in the blood and induce metastasis of cancer cells [22]. Pancreatic cancer forms a suitable pre-metastatic niche in distant organs before metastasis. However, whether pancreatic astrocytes help in the pre-metastatic niche formation is not known. Xu et al. first proved that PSCs are transferred from the primary site of the tumor to distant organs [22], and then Suetsugu et al. found that during pancreatic cancer metastasis, pancreatic cancer cells and pancreatic stellate cells co-metastasize [23]. Following the study on the application of sEVs in pancreatic stellate cells, Yue Feng et al. found that pancreatic cancer cell-derived sEVs promote the recruitment of pancreatic cancer PSCs by activating the LIN28B /let-7/HMGA2/PDGFB signaling pathway through the transfer of the exosomal protein LIN28B to recipient cells [24]. Pancreatic cancer cell-derived sEVs promote the expression of ACTA2 and fibrosis-related genes in PSCs, and form a niche inflammatory environment by stimulating the expression of miR-1246 and miR-1290 [25]. sEVs released from pancreatic cancer recruit pancreatic stellate cells to the site of tumor metastasis through the bloodstream and promote the formation of pre-metastatic niches. Pancreatic stellate cell-derived sEVs can also act on the microenvironment of pancreatic cancer cells to promote the development and metastasis of pancreatic cancer cells. Takikawa et al. showed that pancreatic stellate cell-derived sEVs promote proliferation and metastasis of pancreatic cancer cells [26]. sEVs differentially alter the expression of genes that regulate cancer cell processes, including DNA replication and repair, cell cycle, and cell death, as well as cell proliferation and metastasis within tumor cells [27]. However, the RNA content of sEVs is the most critical factor affecting the progression of pancreatic cancer. PSC-derived sEVs miR-5703 can target CMTM4 in PC cells, and activate PI3K/Akt pathway through PAK4 to promote the proliferation of cancer cells [14]. High expression of miR-21-5p and miR-451A, can stimulate the proliferation and metastasis of pancreatic cancer cells [26]. PSC-derived sEV miR-21 also promotes PDAC cell migration and EMT, and enhance RAS/ERK signaling activity to regulate a series of biological responses in pancreatic cancer cells [13]. The RNA components of sEVs also play an active role in some special environments. Hypoxia can up-regulate the expression of miR-4465 and miR-616-3p in PSC-derived sEVs, and these miRNAs promote PC progression and metastasis by inhibiting the PTEN/ Akt pathway [28]. Pancreatic cancer cells interact with pancreatic stellate cells to reshape a new tumor microenvironment to promote the metastasis of pancreatic cancer (Fig. 1).

### A suitable pre-metastatic niche is formed in the distant organs

#### sEVs promote angiogenesis in distant organs during metastasis

**Fig. 2:**
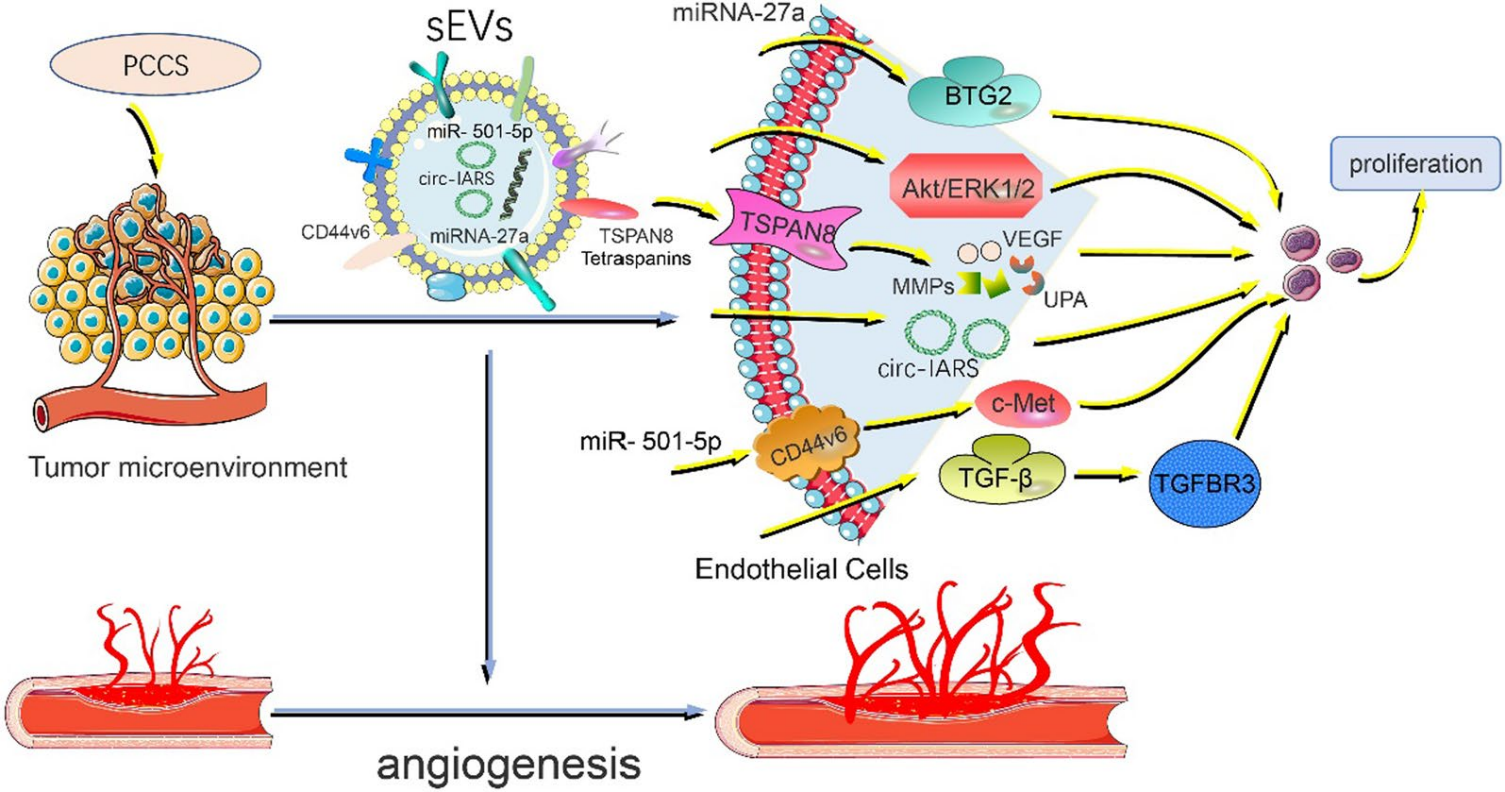
VEGF: vascular endothelial growth factor, MMPs: matrix metalloproteinases, UPA: urokinase-type plasminogen activator, HUVECS:Human umbilical vein endothelial cells. Mechanisms by which sEVs promote angiogenesis when secreted to distant organs. When sEVs reach distant organs, TSPAN8 and other four asppanins carried on them can enhance the expression of pro-vascular factors such as VEGF, MMPs and UPA. Secondly, miRNA, circ-iars and other components carried by pancreatic cancer-derived sEVs promote angiogenesis by inducing HMVEC growth. In addition, non-tumor cell-derived sEVs can also promote angiogenesis. TAMS-derived sEVs promote angiogenesis by activating the TGF-β signaling pathway and down-regulating TGFbR3

While normal angiogenesis is critical for development and tissue growth, pathological angiogenesis supports the growth and spread of cancers by supplying nutrients and oxygen, and providing a conduit for distant metastasis [29]. Pancreatic cancer vascularization is characterized by a high microvascular density, impaired microvessel integrity and poorly perfused vessels with heterogeneous distribution [30]. sEVs promote angiogenesis during, cancer progression by transporting numerous pro-angiogenic biomolecules like vascular endothelial growth factor (VEGF), matrix metalloproteinases (MMPs), and microRNAs [31]. sEVs labeled TSPAN8 and other tetraspanins (e.g., CD9 and CD63) are highly expressed in pancreatic cancer cells. On being secreted to metastatic organs, sEVs upregulate the expression of VEGF, increase the secretion of MMPs, and promote urokinase-type plasminogen activator (UPA) to promote angiogenesis [32, 33]. sEVs carrying CD44V6, which is highly expressed in pancreatic cancer, promote angiogenesis by activating c-Met and facilitate pre-metastatic niche formation [34]. RNA carried by pancreatic cancer-derived sEVs also promote angiogenesis in distant organs. Pancreatic cancer cell-derived sEVs, carrying miRNA-27a, promote HMVEC angiogenesis through BTG2 in preparation for tumor metastasis [35]. Pancreatic cancer PK-45H cells promote angiogenesis through the release of sEVs by dynamic dependent endocytosis of Human umbilical vein endothelial cells (HUVECs) and phosphorylation of Akt and ERK1/2 signaling molecules [36]. Li et al. also found that pancreatic cancer-derived exosomal Circular RNA IARS (circ-iars) promoted angiogenesis and tumor metastasis by enhancing the permeability of endothelial monolayer and inducing HUVEC growth [37]. sEVs released by other cells also contribute to angiogenesis. sEVs secreted by TAMS downregulate TGFbR3 by activating the TGF-β signaling pathway and promote angiogenesis in distant organs to provide an environment suitable for the survival of pancreatic cancer tumor cells [38]. sEVs promote the generation of blood vessels in distant organs by regulating vascular growth factors and HUVECs of target organs, thus preparing suitable pre-metastatic niches for tumors (Fig. 2).

#### sEVs are involved in remodeling the extracellular matrix of pre-metastatic niches

The alteration of the extracellular matrix is a key factor in the formation of pre-metastatic niche, and the remodeled extracellular matrix creates a suitable environment for the seeding and growth of Circulating tumor cell (CTC) [39]. Proteins and other RNAs carried on the surface of sEVs play an important role in changing the extracellular matrix and such changes are mainly manifested by inflammation, fibrosis, and damage of the extracellular matrix and prepare the distant organs for metastasis by tumor cells. In a mouse model, Kupffer cells untake of PDAC-derived sEVs with MIF in the liver causes these macrophages to release TGF-β, which in turn promotes fibronectin expression in hepatic stellate cells. This ECM remodeling of hepatic stellate cells allowed macrophages to be recruited from bone marrow and induced the formation of a pre-metastatic liver niche [40]. PMN formation is impeded when sEV-mediated extracellular matrix remodeling is blocked. Yue et al. found that CD151 and Tspan8 carried by sEVs were directly related to integrins and proteases. The depletion of CD151 and Tspan8 secreted by sEVs affected the degradation defects of ECM. It leads to exosomal secretion-mediated PMN formation injury, which was not conducive to tumor metastasis [41]. Further, sEVs of pancreatic tumor cells were isolated from pancreatic cancer patients and tenascin C was found to be a highly abundant protein in pancreatic cancer-derived sEVs, tenascin C can bind to other members of the ECM protein and cell surface receptors and play a key role in tissue remodeling [42]. Specific sEV integrins also interact with extracellular matrices, and the deposited laminin and fibronectin may help increase the adhesion of extracellular matrices and facilitate colonization of circulating tumor cells [43].The remodeling, deposition, and cross-linking of the extracellular matrix ultimately leads to the development of fibrosis that hardens the matrix and promotes the growth of malignancy [44]. sEVs remodel the extracellular matrix by carrying growth factors, chemokines, miRNAs and other components to provide an appropriate pre-metastatic niche for pancreatic cancer cell metastasis. However, the dense extracellular matrix can reduce the adhesion of cancer cells and recruit cancer cells for metastasis to organs through inflammatory factors.

#### sEVs mediate the formation of immunosuppressive environments in distant organs.

We concluded that macrophages, bone marrow suppressor cells, natural killer cells, and neutrophils play a major role in innate immunity in pancreatic cancer. Among them, m1-type macrophages and natural killer cells are involved in anti-tumor immunity, while bone marrow suppressor cells contribute to immune evasion. Recent studies have shown that M2 macrophages promote immune evasion of PDAC by producing anti-inflammatory signals. In adaptive immunity to PDAC, T cells play a key anti-tumor role, while B cells play a supplementary role. On being recruited to the tumor microenvironment, the macrophages become the tumor-associated macrophage, by producing cytokines, growth factors, such as VGEF, to promote angiogenesis and immunosuppression, Inhibition of TAMs, increases the recruitment of T cell immune penetrating [45]. MDSCs recruited by tumor cells inhibit the function of T cells by regulating PD-L1 [46]. Pancreatic cancer cells inhibit NK cell activity by direct toxicity to NK cells and by secretion of indoleamine 2,3-dioxygenase (IDO)、 IL-10 and TGF-β [47]. At distant metastatic sites, pancreatic cancer cells recruit bone-derived suppressor cells via EVs and inhibit regulatory T cell function via TGF-β, IL-10, and IFN-γ [48, 49]. Neutrophils are also recruited to neutralize other immune cells through IL-12 and TNF-α [45]. Tumor cell-derived sEVs inhibit the function of the immune cells, such as macrophages, NK cells, T cells, B cells, and help in the immune escape of tumor cells [50–54]. This suppression of immune cell function also provides an immunosuppressive environment for the pre-metastatic niche of tumor metastasis. M2 macrophage-derived sEVs in pancreatic cancer overexpress Lncrna sBF2-AS1, which competes for endogenous RNA in vivo. Inhibition of Mir-122-5P and up-regulation of XIAP promote PC proliferation [55]. Pancreatic cancer-derived sEVs induce ER stress-mediated apoptosis of T lymphocytes through the p38 MAPK pathway [56].Pancreatic cancer-derived sEVs downregulate TLR4 and downstream cytokines in dendritic cells(DCs) by miR-203, thereby inhibiting the immune response [57]. Pancreatic cancer-derived sEVs inhibit the expression of regulatory factor X-associated protein(RFXAP) through miR-212-3p, thereby reducing the expression of MHC II, inducing immune tolerance of dendritic cells, and inhibiting the immune response [58]. Another sEV-mediated mechanism that helps cancer cells evade the immune effector cells is the use of the decoy [59]. sEVs serve as targets for B cells in pancreatic ductal carcinoma and act as a decoy for complement-mediated cytotoxicity, thereby preventing an immune response against the pancreatic cancer cells [60]. Tumor sEVs also promote the formation of an immunosuppressive environment in the pre-metastatic niche through the recruitment of immunosuppressive cells [43, 61].Macrophage migration inhibitors are highly expressed in pancreatic cancer-derived sEVs, which recruit marrow-derived macrophages to induce pre-metastatic niche formation in the liver [40]. It is also possible to reprogram the recruited immune cells into immunosuppressive myeloid cells in the pre-metastatic niche, thereby inhibiting anti-tumor immunity [62]. Not all tumor sEVs are immunosuppressive. some tumor-derived sEVs also induce a PMO-dependent innate immune response to promote immune cell-medicated clearance of cancer cells [63]. The expression of miR-128 on pancreatic cancer tumor cells increases the number of dendritic cells, CD8 + T lymphocytes and natural killer T cells (NKT) in the tumor and spleen, thereby improving anti-tumor immunity [64]. In the future, if sEVs are isolated and accurately, they may be a potential target for the treatment of pancreatic cancer. Understanding the mechanism of exosomal mediated immunosuppression is necessary (Table1) and will help in developing new therapeutic strategies in pancreatic cancer.

**Table 1:** The role of different sEVs in the tumor microenvironment with corresponding immune cell populations

sEVs	Acting cell	Role in the formation of immunosuppressive environments	References
High expression of miR—203	Dendritic cells	Down-regulation of TLR4 and downstream cytokines, thereby inhibiting the immune response	[57]
High expression of miR-212-3p	Dendritic cells	Inhibit the expression of regulatory factor X-associated protein (RFXAP), reduce the expression of MHC II, and produce immune tolerance	[58]
High expression of CD63	T lymphocyte	Activate p38 mitogen-activated protein kinase (MAPK), induce cell apoptosis, and eventually lead to immunosuppression	[56]
High expression of tumor-associated antigens (TAAs)	B cell	As a bait for complement, it produces cytotoxicity and inhibits specific immune response	[60]
High expression of macrophage migration inhibitory factor (MIF)	Bone marrow-derived macrophages	Immunosuppressive cells are recruited to form an immunosuppressive environment	[40]
High expression of miR153	NK cells	Natural killer group 2 member D (NKG2D) was reduced by upregulation of hypoxia inducible factor 1-α (Hi1FA), and NK cells cleavage	[79]
High expression of miR-338-3p	Neutrophils	At present, the mechanism of action is not clear, biochemistry analysis indicated that it can inhibit the function of immune cells	[80]
High expression of miR-199b-5p	Neutrophils	At present, the mechanism of action is not clear, biochemistry analysis indicated that it can inhibit the function of immune cells	[80]
Lower expression of miR-340	Macrophages	At present, the mechanism of action is not clear, inhibits macrophages from becoming M1-like phenotype polarization in the peripheral and tumor immune microenvironment, and reduces T cells, especially CD8 + T cells	[81]
Lower expression of miR-128	All kinds of immune cells	At present, the mechanism of action is not clear, biochemistry analysis indicated that it can inhibit the function of immune cells	[64]

#### sEVs mediate localized metastasis of pancreatic cancer to specific organs

**Fig. 3:**
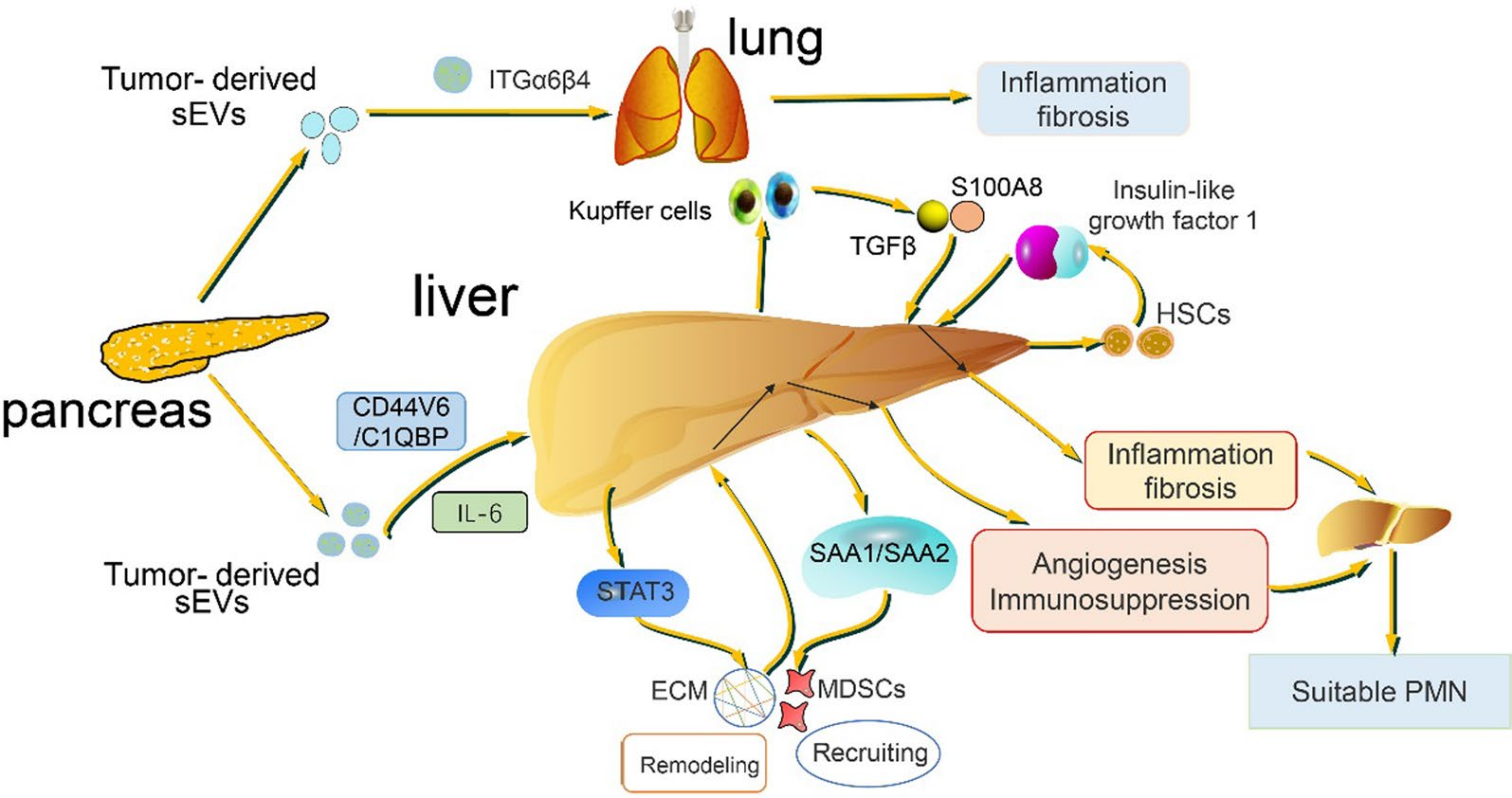
Mechanism of targeted metastasis of pancreatic cancer to liver and lung. Upon arrival in the liver, sEVs derived from pancreatic cancer first express the integrin αVβ5 they carry, which promotes the release of pro-inflammatory factor S100A8 by activating Kupffer cells, making the liver an inflammatory environment. The CD44v6/C1QBP complex carried by sEVs also has the same mechanism by phosphorylating insulin-like growth factor 1 signaling molecules, thereby initiating the development of liver fibrosis. Secondly, the sEVs secreted by fibroblasts promote the expression of serum amyloid protein and STAT3 by releasing the pro-inflammatory factor IL-6 and binding to the IL-6 receptor on the liver, and the liver becomes an inflammatory and immunosuppressive environment by recruiting immunosuppressive cells. SEVs ITGα6β4 and ITGα6β1 bind to lung cells and epithelial cells and regulate lung specific metastasis by forming an inflammatory environment in the lung

Tumor metastases to organs have been a puzzle since Stephen Paget proposed the "seed and soil" hypothesis in 1889 [65]. The localized metastasis of tumor attracts people's continuous exploration, and studies in recent decades have shown that in the process of tumor metastasis, tumor is like a seed, and sEVs play the role of "bridge" and "digger", on the one hand, promoting tumor cells to detach and metastasize from the primary site, and on the other hand, forming a pre-metastatic niche suitable for tumor growth in distant organs [66–70]. The concept of "organotropism" is well documented in breast cancer [71, 72]. 76–80% of patients with pancreatic cancer have liver metastasis; Other common sites of metastasis included peritoneum (48%) and lung (45%) [73, 74]. It has been proposed that pancreatic cancer-derived sEVs induce TGFβ secretion through activation of Kupffer cells and recruit myeloid suppressor cells, making the liver one of the most suitable sites for pancreatic cancer metastasis [40]. sEVs secreted by pancreatic duct carcinoma express integrin αVβ5, which, when absorbed by liver Kupffer cells, release pro-inflammatory S100A8 and promote pancreatic cancer metastasis to the liver, while sEVs ITGα6β4 and ITGα6β1 bind to lung cells and epithelial cells and regulate lung specific metastasis [69]. Proinflammatory cytokines secreted by pancreatic fibroblasts also play an important role in the localized metastasis of pancreatic cancer to the liver. sEVs promote STAT3 expression and serum amyloid A1 (SAA1) and SAA2 secretion by transporting the pro-inflammatory, cytokine IL-6 secreted by the fibroblasts, from pancreatic tumors to the IL-6 receptor on liver cells. As a result, the liver begins to activate the deposition of the extracellular matrix and to recruit myeloid suppressor cells to suppress the immune response, all of which provide a perfect pre-metastatic niche for pancreatic cancer cells to metastasize to the liver [75]. Fibrosis in the liver may be used to explain localized metastases as the inflammatory environment develops further. The sEV CD44V6 /C1QBP complex is delivered to the plasma membrane of hepatic satellite cells (HSCs), leading to phosphorylation of insulin-like growth factor 1 signaling molecules, which leads to HSCs activation and liver fibrosis, providing an appropriate pre-metastatic niche for subsequent pancreatic cancer metastasis to the liver [76]. As for localized metastasis of pancreatic cancer, based on the role of sEVs, it is more likely to metastasize to distant target organs such as the lung and liver (Fig. 2). As for the localized metastasis of pancreatic cancer to the liver, this may also be because of the central role of the liver in metabolism [77]. The study of Kupffer cells, especially protein and phosphorylation, can be used to provide more insights into the metastasis of pancreatic cancer to the liver [78]. The driving factors for the specific metastasis of pancreatic cancer to the liver site are mainly inflammatory factors and immunosuppressive factors produced from the damaged part of the liver, etc. They interact with the cancer cells at the primary tumor site, greatly reducing the adhesion of cancer cells, enhancing their blood circulation and circulation to the liver, and ultimately making the pancreatic cancer cells more inclined to localize to the liver site. The study related to sEVs will facilitate us to further reveal the mystery of localized metastasis of pancreatic cancer to specific organs (Fig. 3).

## Future prospects of sEVs in pancreatic cancer

### sEVs can be used as early markers for the diagnosis of pancreatic cancer

The molecules carried by sEVs can be used as biomarkers for diagnosis or prediction of cancer since sEVs carry unique DNA, RNA, microRNAs and lncRNAs, proteins and lipids, and display specific gene expression profiles [82]. sEVs are widely found in human plasma and other components, and quantitative analysis of miRNAs and other molecules carried by sEVs can be used to detect whether pancreatic cancer has a pre-metastatic niche that is conducive to metastasis. The sEVs miRNA-1246 and miRNA196-a [83], two highly enriched miRNAs in pancreatic cancer sEVs, show significantly different expression in localized and metastatic pancreatic cancer. The high expression of miRNA-1226 may induce cell death by inhibiting the expression of MUC1 oncoprotein, disrupt the surrounding microenvironment of cancer cells, and inhibit the metastasis of cancer cells [84]. Low expression of miRNA-214 and miRNA-508 in sEVs may also inhibit the formation of fibrosis, and is associated with a better prognosis in pancreatic cancer [85]. CircRNA PDE8A in sEVs can promote the occurrence and development of pancreatic cancer through Mir-338 /MACC1/MET pathway, which has a significant role in the progression of pancreatic cancer and can be as a potential biomarker [86]. sEV proteins are also used as markers for the diagnosis of pancreatic cancer. Melo et al. found that the levels of glypican-1 (GPC1) in sEVs of patients with early pancreatic cancer were significantly higher than those of normal subjects, while the diagnostic rate was extremely high in the early and late diagnosis of pancreatic cancer, which could be a very meaningful research direction [87]. CKAP4 secreted in sEVs [88] and CD63 and CD9 secreted in [89] have great potential in the diagnosis of pancreatic cancer. The specificity and sensitivity of EphA2 in sEV as a possible biomarker has shown to be extremely high and is of great significance in the diagnosis of patients with pancreatic cancer and pancreatitis [90]. In addition, sEV carrying members of the EGFR, EpCAM, HER2, MUC1, and Wnt families are also highly sensitive and specific in the diagnosis of pancreatic cancer and can be used as early biomarkers [91]. Here, we will summarize the mechanisms of different sEVs in the diagnosis of pancreatic cancer (Table 2).

**Table 2:** Candidate biomarkers of sEVs for early detection of PC and their application

Sources	sEVs	Application in pancreatic cancer	References
hPaCa	CD63, CD9	Diagnosis:to distinguish whether the pancreas has malignant lesions	[89]
hPaCa	CD44v6	Diagnosis:promote metastasis and invasion	[104]
mPaCa	CD151/Tspan8	Diagnosis:promote metastasis and angiogenesis	[105]
hPaCa	CKAP4	Diagnosis:promote proliferation and migration	[88]
hPaCa	miRNA-10b	Diagnosis:distinguish between patients with pancreatitis and those with pancreatic cancer	[106]
hPaCa	miRNA-1226-3p	Diagnosis: promote proliferation and migration	[84]
hPaCa	miRNA-214,miRNA-508	Diagnosis:to indicate high or low survival rate	[85]
hPaCa	miRNA-4525,miRNA-21	Diagnosis: to identify patients at high risk of recurrence after excision	[107]
hPaCa	miRNA-16a,miRNA-196a	Diagnosis:to distinguish pancreatic cancer from pancreatitis and healthy people	[108]
hPaCa	miRNA-27a	Diagnosis:promote invasion and angiogenesis	[109]
hPaCa	miRNA-301a-3p	Diagnosis: promote the invasion	[110]
mPaCa	miRNA-339-5P	Diagnosis:promote invasion and metastasis	[111]
hPaCa	miRNA-17-5p	Diagnosis: to distinguish between pancreatic cancer patients	[112]
hPaCa	miRNA-483-3p	Diagnosis: distinguish pancreatic cancer from other tumors	[113]
hPaCa	miRNA-550	Diagnosis: distinguish pancreatic cancer from other tumors	[114]
macrophage	miRNA-501-3p	Diagnosis:promote tumor metastasis and development	[15]
hPaCa	miRNA-191,miRNA-21	Diagnosis:distinguish pancreatic cancer from other tumors	[115]
hPaCa	miRNA-143	Diagnosis:inhibits metastasis and proliferation	[116]
hPaCa	miRNA-33b	Diagnosis:promotes proliferation and migration	[117]

*hPaCa* human pancreatic cancer cells, *mPaCa* mouse pancreatic cancer cells

### Now and future: sEVs for pancreatic cancer treatment

The main reason why pancreatic cancer is resistant to chemotherapy is because of the microenvironment surrounding pancreatic cancer cells, especially pancreatic stellate cells, which prevent drug entry into pancreatic cancer. As endogenous extracellular vesicles, sEVs can carry a variety of miRNAs and other components, and are considered natural nanoscale delivery agents and have great potential in drug delivery [92]. sEVs can pass directly through the microenvironment surrounding pancreatic cancer without being blocked by pancreatic stellate cells. Mesenchymal stromal cells (MSCs) can target the tumor microenvironment and secrete sEVs in large amounts. Wrapped in MSCs, sEVs containing paclitaxel (PTX) can be delivered to pancreatic cancer, preventing the obstruction of MSCs by surrounding tumor cells. sEVs containing doxorubicin [93] and sEVs carrying curcumin [94] were designed to mediate cytotoxicity of pancreatic cancer cells and accelerate the death of cancer cells, and both have shown good efficacy in the treatment of pancreatic cancer. sEVs in pancreatic cancer also inhibit the metastasis of pancreatic cancer by miRNAs and proteins carried by some sEVs. The sEV miR-410-3p enhanced the sensitivity of PDAC cells to gemcitabine and reduced drug resistance by inhibiting autophagy in HMGB1-induced PDAC cells during chemotherapy [95]. MiR-210 produces inactivated mesenchymal pancreatic stellate cells (PSCs), which facilitate drug delivery to the tumor site of pancreatic cancer [96]. sEVs can also carry curcumin to act on pancreatic cancer cells and promote cytotoxicity in pancreatic cancer cells [97]. sEV in pancreatic cancer is not only to promote the development of pancreatic cancer, metastasis [98].Some sEVs carry miRNAs and proteins that can also inhibit pancreatic cancer metastasis. sEV miR-7 inhibits PC cell proliferation and induces apoptosis by directly targeting MAP3K9 [99], and miR-195 mediate tumor-suppressive effects in PC by targeting DCLK1 [100].

A comprehensive understanding of sEVs, especially the proteins, miRNAs and other substances carried on sEVs in need since. These specific substances may give insights into the development, and metastasis of pancreatic cancer. A meta-analysis by Zhu et al. showed that liquid biopsy is the best for the diagnosis of pancreatic cancer, while sEVs higher specificity and sensitivity compared with liquid biopsy methods such as ctDNA and CTC [101]. In addition, miRNAs [102] and lncRNAs [103] carried by sEVs are the most promising biomarkers in the diagnosis of pancreatic cancer. Currently, although the use of sEVs remains largely theoretical, ongoing clinical trials will reveal the potential of sEVs in pancreatic cancer development and therapy. We reviewed clinical trials of sEVs in pancreatic cancer in recent years (Table 3).

**Table 3:** FDA-approved exosomes in clinical trials for the treatment of pancreatic cancer

NCT number	Title	Status	Conditions	Interventions	Phases
NCT03821909	Acquisition of Portal Venous CTCs and Exosomes From Patients With Pancreatic Cancer by EUS	Unknown status	Pancreatic Cancer	Procedure: Endoscopic ultrasound-guided protal venous blood sampling	
NCT02393703	Interrogation of Exosome-mediated Intercellular Signaling in Patients With Pancreatic Cancer	Recruiting	Pancreatic Cancer|Benign Pancreatic Disease		
NCT03608631	iExosomes in Treating Participants With Metastatic Pancreas Cancer With KrasG12D Mutation	Recruiting	KRAS NP_004976.2:p.G12D|Metastatic Pancreatic Adenocarcinoma|Pancreatic Ductal Adenocarcinoma|Stage IV Pancreatic Cancer AJCC v8	Drug: Mesenchymal Stromal Cells-derived Exosomes with KRAS G12D siRNA	Phase 1
NCT03032913	Diagnostic Accuracy of Circulating Tumor Cells (CTCs) and Onco-exosome Quantification in the Diagnosis of Pancreatic Cancer—PANC-CTC	Completed	Pancreatic Ductal Adenocarcinoma (PDAC)	Pancreatic Ductal Adenocarcinoma (PDAC)	
NCT03711890	Ultra-High Resolution Optical Coherence Tomography in Detecting Micrometer Sized Early Stage Pancreatic Cancer in Participants With Pancreatic Cancer	Recruiting	Pancreatic Carcinoma|Pancreatic Intraductal Papillary Mucinous Neoplasm, Pancreatobiliary-Type	Procedure: Optical Coherence Tomography|Procedure: Therapeutic Conventional Surgery|Diagnostic Test: Laboratory Evaluation	Not Applicable
NCT03791073	New Biomarkers in Pancreatic Cancer Using EXPEL Concept	Active, not recruiting	Oncology		
NCT03250078	A Pancreatic Cancer Screening Study in Hereditary High Risk Individuals	Recruiting	Pancreatic Neoplasms	Diagnostic Test: MRI/MRCP	
NCT04636788	Circulating Extracellular Exosomal Small RNA as Potential Biomarker for Human Pancreatic Cancer	Recruiting	Pancreas Adenocarcinoma	Procedure: Venous sampling	Not Applicable
NCT03410030	Trial of Ascorbic Acid (AA) + Nanoparticle Paclitaxel Protein Bound + Cisplatin + Gemcitabine (AA NABPLAGEM)	Recruiting	Pancreatic Cancer|Pancreas Cancer|Pancreatic Adenocarcinoma Resectable|Pancreatic Ductal Adenocarcinoma|Pancreas Metastases	Drug: Ascorbic Acid|Drug: Paclitaxel protein-bound|Drug: Cisplatin|Drug: Gemcitabine	Phase 1|Phase 2
NCT03334708	A Study of Blood Based Biomarkers for Pancreas Adenocarcinoma	Recruiting	Pancreatic Cancer|Pancreatic Diseases|Pancreatitis|Pancreatic Cyst	Diagnostic Test: Blood Draw|Diagnostic Test: Tumor Tissue Collection|Diagnostic Test: Cyst Fluid	

Although the research on sEVs has increased, the application of sEVs in clinical practice remains a challenge. A completed clinical trial (NCT03032913) has shown promising results in the diagnosis of pancreatic cancer using sEVs.However, the application of sEVs in the treatment of pancreatic cancer is still in the early stages.A clinical trial (NCT03608631) used sEVs for the treatment of metastatic pancreatic cancer with a KRASG12D mutation.At present, there has not been much progress in this kind of research, but the application of sEVs as designed drug carriers in the treatment of pancreatic cancer is a big breakthrough, and may become a new approach for the treatment of pancreatic cancer in the future.

## Conclusion

In this review, we summarize the two major steps involved in exosomal mediated metastasis of pancreatic cancer, focusing on the mechanisms by which sEVs mediate the interaction between pancreatic cancer cells and their surrounding microenvironment. Cancer cells act on distant organs by releasing sev, which eventually constructs a pre-metastatic niche suitable for tumor cell metastasis by promoting angiogenesis, remodeling the extracellular matrix and forming an immunosuppressive microenvironment in distant organs. Starting from the entry point to the distant microenvironment is conducive to a more intuitive understanding of the process of pancreatic cancer metastasis. At the same time, we also summarized the mechanism of localized metastasis of pancreatic cancer to some organs. Finally, in the direction of microenvironment-based research, we summarized the possibility of using sEVs as biomarkers for the early diagnosis of pancreatic cancer and the possibility of using sEVs directly or as drug carriers for the treatment of pancreatic cancer. These findings will provide a new and promising direction for research on the diagnosis and treatment of pancreatic cancer metastasis.

## Data Availability

Not applicable.

## Abbreviations

CAF: Cancer associated fibroblasts
CTC: Circulating tumor cell
DC: Dendritic cells
ECM: Extracellular matrix
EMT: Mesenchymal transition
ESCRT: Endosomal sorting complex required for transport
HSCs: Hepatic satellite cells
HUVECs: Human umbilical vein endothelial cells
GPC1: Glypican-1
MMPs: Matrix metalloproteinases
MIF: Migration inhibitory factor
MSCs: Mesenchymal stromal cells
NKT: Natural killer T cells
PMN: Pre-metastatic niche
PSC: Pancreas stellate cells
RFXAP: Regulatory factor X-associated protein
EVs: Small extracellular vesicles
TAMs: Tumor associated macrophage
TGFβ: Transforming growth factor-β
TME: Tumor micro-environment
UPA: Urokinase-type plasminogen activator
VEGF: Vascular endothelial growth factor
